# Learning-Based Lane-Change Behaviour Detection for Intelligent and Connected Vehicles

**DOI:** 10.1155/2020/8848363

**Published:** 2020-09-30

**Authors:** Luyao Du, Wei Chen, Zhonghui Pei, Hongjiang Zheng, Shuaizhi Fu, Kang Chen, Di Wu

**Affiliations:** ^1^School of Automation, Wuhan University of Technology, Wuhan 430070, China; ^2^School of Information Engineering, Wuhan University of Technology, Wuhan 430070, China; ^3^Shanghai Engineering Technology Research Center for Intelligent and Connected Vehicle Terminals, Shanghai 200030, China; ^4^Shanghai PATEO Electronic Equipment Manufacturing Co., Ltd., Shanghai 200030, China; ^5^Key Laboratory of Environment Change and Resources Use in Beibu Gulf, Nanning Normal University, Ministry of Education, Nanning 530001, China; ^6^GNSS Research Center of Wuhan University, Wuhan 430000, China

## Abstract

Detection of lane-change behaviour is critical to driving safety, especially on highways. In this paper, we proposed a method and designed a learning-based detection model of lane-change behaviour in highway environment, which only needs the vehicle to be equipped with velocity and direction sensors or each section of the highway to have a video camera. First, based on the Next Generation Simulation (NGSIM) Interstate 80 Freeway Dataset, we analyzed the relevant features of lane-changing behaviour and preprocessed the data and then used machine learning algorithms to select the suitable features for lane-change detection. According to the result of feature selection, we chose the lateral velocity of the vehicle as the lane-change feature and used machine learning algorithms to learn the lane-change behaviour of the vehicle to detect it. From the dataset, continuous data of 14 vehicles with frequent lane changes were selected for experimental analysis. The experimental results show that the designed KNN lane-change detection model has the best performance with detection accuracy between 89.57% and 100% on the selected dataset, which can well complete the vehicle lane-change detection task.

## 1. Introduction

Over the past few years, with the rapid development of artificial intelligence and communication technology, intelligent vehicles based on intelligence and networking have become a major trend in the development of the automotive industry. From the perspective of technological development, intelligent vehicles are divided into three development directions: connected vehicle (CV), autonomous vehicle (AV), and the integration of the former two, namely, connected and automated vehicle (CAV) or intelligent and connected vehicle (ICV) [1].

ICVs play an important role in improving driving safety and reducing driver burden, contribute to energy conservation and environmental protection, and improve traffic efficiency. Research shows that, in the initial stage of ICVs, advanced intelligent driving assistance technology can help reduce traffic accidents by about 30%, improve traffic efficiency by 10%, and reduce fuel consumption and emissions by 5% [2]. Entering the ultimate stage of the ICVs, that is, the fully automatic driving stage, it could avoid traffic accidents, improve traffic efficiency, and finally liberate people from boring driving tasks.

Driving behaviour detection plays a significant role in ICVs' decision-making system. During the driving of the vehicle, due to the driver's lack of attention or the obstruction of the surrounding large vehicles, it is likely that the driver will not be able to fully understand the driving conditions of the surrounding vehicles, thus causing great safety risks. Many methods of lane-change behaviour detection have been proposed by researchers in recent years, including hidden Markov model (HMM) [3–5], multi-view convolutional neural network model (MV-CNN) [6], and vision-based deep residual neural network (RNN) [7]. Detection of lane-changing behaviour in different scenarios, including highways [8–12] and signalized intersections [13–16], has also been studied by many researchers. Steering behaviour recognition [17] and prediction [18] methods have been proposed, too. Besides, some new deep learning and machine learning methods have also been proposed in recent years. Xie et al. comprehensively modeled lane-change using deep learning approaches including deep belief network and long short-term memory [19]. Xing et al. proposed an ensemble bi-directional LSTM model for driver intention inference [20]. Gao et al. proposed a data-driven lane-change detection system using deep learning techniques [21]. Zhang et al. modeled the car following and lane-changing behaviours simultaneously using hybrid retraining constrained long short-term memory neural networks [22]. Zhao et al. proposed a new quantitative discriminant model based on deep belief networks algorithm and the classification analysis method based on support vector machine [23]. Dang and Dai established a lane-change model based on improved Bayesian network [24]. These methods, however, need prior knowledge, or the structure is complex and the real-time performance can be improved. In practical application scenarios, there is usually lack of prior knowledge of data distribution, and a simpler classification method is easier to implement.

In this paper, we proposed a method and designed a learning-based detection model of lane-change behaviour on highways, which only needs the vehicle to be equipped with velocity and direction sensors or each section of the highway to have a video camera. The main contributions of this paper can be summarized as follows:

1. Based on the NGSIM Interstate 80 Freeway Dataset, the vehicle lane-change behaviour characteristics were analyzed and selected, and the data, including non-lane-change, single lane-change, and sequential lane-change, was preprocessed and reconstructed.
2. Based on the analysis of the vehicle lane-change process, and considering the real-time requirements in the application of ICVs, the vehicle lane-change detection model based on K-Nearest Neighbor (KNN) is proposed and compared with extra tree (ET) and random forest (RF).
3. Through feature selection, the lateral speed, which is combined with speed and driving direction and is easy to be collected, is excavated as a feature for lane-change detection. The continuous data of 14 vehicles with frequent lane changes were tested and analyzed experimentally and performed well with accuracy between 89.57% and 100% on lane-change behaviour detection.

The rest of the paper is organized as follows.  Section 2 explains the details of the dataset.  Section 3 describes the methodology of lane-change behaviour detection, including feature selection and lane-change detection method.  Section 4 presents the experiments and results of lane-change behaviour detection.  Section 5 concludes this paper and discusses the future work.

## 2. Data Processing

**Figure 1:**
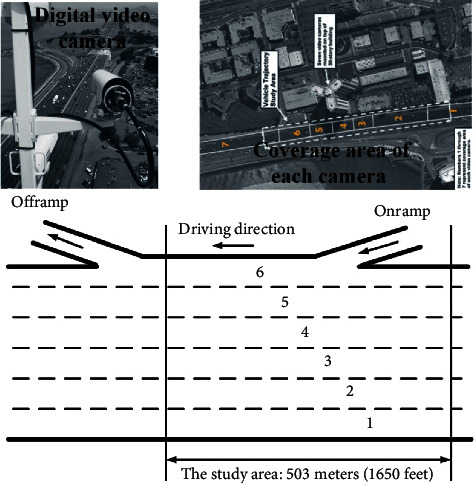
The collection scene description of data. The six-lane study area, which is divided into seven sub-areas, is photographed and recorded by digital video cameras.

In order to verify the lane-change detection method, NGSIM Interstate 80 Freeway Dataset initiated by the United States Department of Transportation (US DOT) Federal Highway Administration (FHWA), which is freely available at the NGSIM web site at http://ngsim.fhwa.dot.gov, is used and processed. The dataset contains 45 minutes, divided into three periods: 4:00 p.m. to 4:15 p.m.; 5:00 p.m. to 5:15 p.m.; and 5:15 p.m. to 5:30 p.m., which represent the buildup of congestion, the transition between uncongested and congested conditions, and full congestion during the peak period, respectively [25]. As shown in Figure 1, the six-lane study area with a length of 1650 feet is divided into seven sub-areas. In each sub-area, a video detector is installed on the high-rise building near the lane, and the traffic of the sub-area is photographed and recorded.

The original dataset contains many attributes, including some attributes that are not highly relevant to the lane-change detection. In order to establish a dataset suitable for vehicle lane-change detection, the attributes in the dataset that are not highly relevant to lane-change detection were deleted, Vehicle_ID, Lane_ID, V_Length, and V_Width remained the same as those in the original dataset, LX_m, LY_m, Vel_m/s, and Acc_m/s^2^ changed the unit in the original dataset from feet to meters (1 foot = 0.3048 meters), the average lateral velocity of vehicle and instantaneous lateral acceleration of vehicle were, respectively, calculated by LX_m and Acc_m/s^2^ and added to the dataset, and the lane-change behaviour of the vehicle was calculated by Lane_ID, forming a new dataset. In the Lane_changing attribute, 0 means to keep the current lane, 1 denotes a single lane change to the right, −1 stands for a single lane change to the left, 2 represents sequential lane change to the right, and −2 represents sequential lane change to the left. The composition of processed data is shown in Table 1.

The instantaneous lateral acceleration of vehicle Acc*_X* can be calculated as

(1) $$\begin{array}{c} {\text{Acc\_X}}_{t}={\text{Acc\_m/s}}_{t}^{2}*\;\sin\left[\text{acr }\tan\left(\frac{\text{LX}\_{\text{m}}_{t}-\text{LX}\_{\text{m}}_{t-1}}{\text{LY}\_{\text{m}}_{t}-\text{LY}\_{\text{m}}_{t-1}}\right)\right], \end{array}$$

where Acc_X_*t*_ represents the value of Acc_X at time *t*, Acc_m/s_*t*_^2^ denotes the value of Acc_m/s^*2*^ at time *t*, LX_m_*t*_ and LX_m_*t−*1_, respectively, mean the value of  LX_m at times *t* and *t* − 1, while LY_m_*t*_ and LY_m_*t−*1_ stand for the value of LY_m at times *t* and *t* − 1, respectively.

The average lateral velocity of vehicle Vel_X can be calculated as

(2) $$\begin{array}{c} {\text{Vel\_X}}_{t}=\frac{\left(\text{LX}\_{\text{m}}_{t}-\text{LX}\_{\text{m}}_{t-1}\right)}{t}, \end{array}$$

where Vel_X_*t*_ represents the value of Vel_X at time *t*, LX_m_*t*_ means the value of LX_m at time *t*, LX_m_(*t−*1)_ denotes the value of LX_m at time *t* − 1, and *t* is the sampling period of the dataset, which is 0.1 seconds.

Current lane-change behaviour of vehicle Lane_changing can be calculated as

(3) $$\begin{array}{c} \text{Lane}\_{\text{changing}}_{t}=\text{Lane}\_{\text{ID}}_{t}-\text{Lane}\_{\text{ID}}_{t-1}, \end{array}$$

where Lane_changing_*t*_ represents the value of Lane_changing at time *t*, Lane_ID_*t*_ means the value of Lane_ID at time *t*, and Lane_ID_*t*_ denotes the value of Lane_ID at time *t* − 1.

## 3. Methodology

### 3.1. Feature Selection

**Figure 2:**
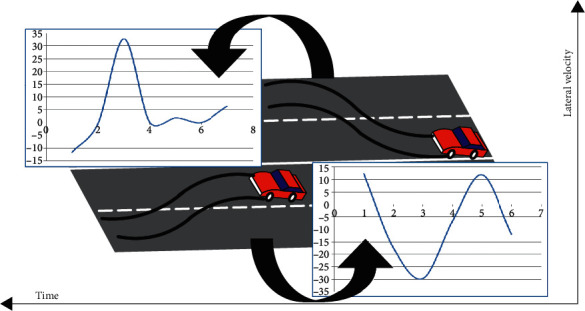
The relationship between lateral velocity and lane change.

In order to accurately detect vehicle lane changes, the relationship between various attributes and vehicle lane changes was analyzed. By analyzing the changing trends of various attributes when the vehicle changes lanes in the dataset, we found that the vehicle's lateral velocity has the most obvious correlation with the lane-change behaviour. The relationship between lateral velocity and lane change is shown in Figure 2, from which we can see that when the vehicle changes lanes, there will be a very obvious change in lateral velocity.

To further analyze and verify the relationship between each attribute and the lane-change behaviour of the vehicle, machine learning models, including KNN [26], extra trees [27], and random forest [28], were performed on each attribute. A total of 27287 lane-changing data were extracted from the dataset. At the same time, in order to balance the number of samples of lane-changing (the moment when the vehicle changes lanes) and non-lane-changing (the moment when the vehicle does not change lanes) data, 27287 non-lane-changing data were selected to form a feature selection dataset. The result of feature selection is shown in Table 2, from which we can see that the detection accuracy with Vel_X as the feature is significantly higher than other features, which can reach more than 90%. In the detection using Vel_X, the accuracy of KNN, extra tree, and random forest is 94.85%, 91.73%, and 92.31%, respectively; KNN has the highest accuracy.

In order to represent the contribution of each feature to the lane-change behaviour detection more intuitively, feature importance analysis, which can be applied to random forest and extra trees, was performed on the dataset. The Gini index was used to measure the feature importance, which can be defined as

(4) $$\begin{array}{c} G_{m}=\sum_{k=1}^{\left|K\right|}\sum_{k^{\prime }\neq k}p_{mk}p_{mk^{\prime }}=1-\sum_{k=1}^{\left|K\right|}p_{mk}^{2}, \end{array}$$

where *K* means that there are *K* categories and *p*_*mk*_ denotes the proportion of category *k* in node *m*. VIM is used to represent the variable importance measures. The importance of feature *X*_*j*_ at node *m*, that is, the change in Gini index before and after the branch of node *m*, can be defined as

(5) $$\begin{array}{c} {\text{VIM}}_{jm}^{\left(\text{Gini}\right)}=G_{m}-G_{\text{l}}-G_{\text{r}}, \end{array}$$

where *G*_l_ and *G*_r_ represent the Gini index of the left and right nodes after the *m* branch. If the node where the feature *X*_*j*_ appears in the decision tree *i* is in the set *M*, then the importance of *X*_*j*_ in the *i*-th tree is

(6) $$\begin{array}{c} {\text{VIM}}_{ij}^{\left(\text{Gini}\right)}=\sum_{m\in M}{\text{VIM}}_{jm}^{\left(\text{Gini}\right)}. \end{array}$$

Assuming there are *n* trees, then

(7) $$\begin{array}{c} {\text{VIM}}_{j}^{\left(\text{Gini}\right)}=\sum_{i=1}{\text{VIM}}_{ij}^{\left(\text{Gini}\right)}. \end{array}$$

Finally, supposing there are *c* features, all the obtained importance scores are normalized:

(8) $$\begin{array}{c} {\text{VIM}}_{j}=\frac{{\text{VIM}}_{j}}{\sum_{i=1}^{c}{\text{VIM}}_{i}}. \end{array}$$

The feature ranking based on feature importance is shown in Table 3, which illustrates that Vel_X has the highest feature importance scores in both random forest and extra tree and is significantly higher than the other six features. Therefore, Vel_X can be selected as a feature of lane-change detection for ICVs.

### 3.2. Lane-Change Detection

#### 3.2.1. Lane-Change Model

The data used in feature selection is discontinuous, so the learned features are relatively independent and have no relationship with the adjacent data. In the actual driving process, the data of vehicle lane-changing behaviour often only takes up a small part of the entire dataset. Therefore, in order to further analyze and establish a lane-change detection model during vehicle driving, we have selected continuous data from 14 vehicles with frequent lane changes for analysis, training, and testing.

**Figure 3:**
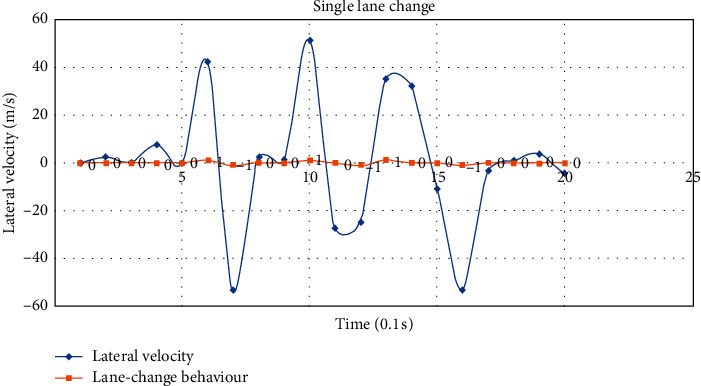
The lateral velocity of single lane change.

**Figure 4:**
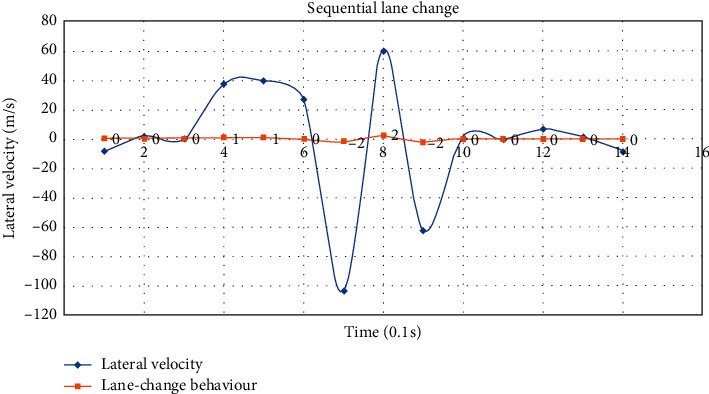
The lateral velocity of sequential lane change.

Lane-change behaviour includes single lane change and sequential lane change. The single lane changes to the left and right are denoted as −1 and 1, while the sequential lane changes to the left and right are denoted as −2 and 2, respectively. The lateral velocity of single lane change intercepted from selected data is shown in Figure 3, from which we can see that there is a significant peak/valley when the vehicle changes lanes, and the threshold of peak/valley can be learned to determine if the vehicle is changing lanes. The lateral velocity of sequential lane change intercepted from selected data is shown in Figure 4; similar to single lane change, there is also a significant peak/valley when the vehicle changes lanes. Besides, there will be a continuous peak/valley or a larger peak/valley of lateral velocity in the sequential lane change, which also can be learned to determine if the vehicle is changing lanes sequentially.

#### 3.2.2. Detection Model

KNN is a simple classification method that can perform effective classification in the absence of prior knowledge of data distribution, and the same are the ET and RF.

RF is an algorithm that integrates multiple trees through the idea of integrated learning and its basic unit is the decision tree [29]. The construction process of the random forest model is mainly divided into four steps:

1. First, a tree needs to be constructed. If there are *N* samples, there are randomly selected *N* samples to be replaced (randomly select one sample at a time, and then return to continue selection). The selected *N* samples are used to train a decision tree as the sample at the root node of the decision tree.
2. Each sample has *M* attributes; when each node of the decision tree needs to be split, *m* attributes are randomly selected from the *M* attributes to satisfy the condition *m* << *M*. Then use some strategy such as information gain and Gini index from the *m* attributes to select one attribute as the split attribute of the node.
3. Repeat step 2 until it can no longer split.
4. Follow steps 1∼3 to build a large number of decision trees to form a forest.

The ET is very similar to RF; they are both composed of many decision trees. The difference is that the RF obtains the best bifurcation attribute in a random subset, while ET obtains the bifurcation value completely randomly, so as to achieve the fork of the decision tree [30].

From the results of feature selection, we can see that when lateral velocity is used as a feature for lane-change detection, KNN has achieved good result, which is better than ET and RF. Therefore, the KNN model is designed using lateral velocity as feature for lane-change detection and the result is compared with ET and RF.

KNN makes predictions using the training dataset directly. Predictions are made for a new data point by searching through the entire training set for the *K* most similar instances (the neighbors) and summarizing the output variable for those *K* instances.

To determine which of the *K* instances in the training dataset are most similar to a new input, a distance measure is used. For real-valued input variables, the Euclidean distance is used as a distance measure method. Euclidean distance is calculated as the square root of the sum of the squared differences between a point a and point *b* across all input attributes *i*.

(9) $$\begin{array}{c} \text{Euclidean distance}\left(a,b\right)=\sqrt{\sum_{i=1}^{n}{\left(a_{i}-b_{i}\right)}^{2}}. \end{array}$$

When KNN is used for classification, the output can be calculated as the class with the highest frequency from the *K*-most similar instances. Each instance in essence votes for their class and the class with the most votes is taken as the prediction.

Class probabilities can be calculated as the normalized frequency of samples that belong to each class in the set of *K* most similar instances for a new data instance. For example, in a binary classification problem (class is 0 or 1),

(10) $$\begin{array}{c} p\left(\text{class}=0\right)=\frac{\text{count}\left(\text{class}=0\right)}{\text{count}\left(\text{class}=0\right)+\text{count}\left(\text{class}=1\right)}. \end{array}$$

The KNN algorithm can be described as follows:

1. Initialize training sets and categories
2. Calculate the Euclidean distance between the test set sample and the training set sample
3. Sort the training set samples in ascending order according to the Euclidean distance
4. Select the first *K* training samples with the smallest Euclidean distance and count their frequency in each category
5. The category with the highest return frequency, that is, the test set sample, belongs to this category

Table 4 shows the step of KNN algorithm, in which the list *I*_*z*_ of its nearest neighbors is determined by calculating the similarity distance between the training object (*x*, *y*)∈*I* and the test object $z=\left(\hat{x},\hat{y}\right)$, where *x* represents the training object, *y* represents the class to which it belongs, and $\hat{x}$ and $\hat{y}$ represent the test object and the class to which it belongs.

## 4. Experimental Results

### 4.1. Evaluation Indicators

When performing machine learning, the confusion matrix of prediction results can be described as shown in Table 5.

The number of pairs of samples divided by the number of all samples is the accuracy (ACC), which can be defined as

(11) $$\begin{array}{c} \text{ACC}=\frac{\text{TP}+\text{FN}}{\text{TP}+\text{TN}+\text{FP}+\text{FN}}. \end{array}$$

Generally, the higher the accuracy, the better the classifier. However, in the case of imbalance between positive and negative samples, the accuracy is a big flaw as an evaluation indicator. It is not scientific and comprehensive to evaluate a model based on accuracy alone.

To evaluate the performance of machine learning more scientifically and comprehensively, precision (*P*), recall (*R*), and *F*1 score can be used.

The precision, which represents the proportion of positive examples that are actually classified as positive examples, can be defined as

(12) $$\begin{array}{c} P=\frac{\text{TP}}{\text{TP}+\text{FP}}. \end{array}$$

The recall, which measures how many positive examples are classified as positive examples, can be defined as

(13) $$\begin{array}{c} R=\frac{\text{TP}}{\text{TP}+\text{FN}}. \end{array}$$

*P* and *R* indicators sometimes conflict, so they need to be considered comprehensively. The most common method is *F*-Measure, which is the weighted harmonic average of *P* and *R*, and can be defined as

(14) $$\begin{array}{c} F=\frac{\left(\alpha^{2}+1\right)P*R}{\alpha^{2}\left(P+R\right)}. \end{array}$$

When the parameter *α* *=* *1*, it is *F*1:

(15) $$\begin{array}{c} F1=\frac{2*P*R}{P+R}. \end{array}$$

*F*1 combines the results of *P* and *R*. When *F*1 is higher, it can indicate that the model is more effective.

In addition, receiver operating characteristic (ROC) curve, in which the abscissa is False Positive Rate (FPR) and the ordinate is True Positive Rate (TPR), is also an important evaluation indicator. The definition of TPR is the same with *P*, and the FPR can be defined as

(16) $$\begin{array}{c} \text{FPR}=\frac{\text{FP}}{\text{TN}+\text{FN}}. \end{array}$$

The area under the ROC curve is called AUC. The prediction effect of a classification model can be evaluated based on the AUC value; the larger the AUC value, the better the performance of the model.

### 4.2. Experiments and Analysis

The model is built on vscode using python and uses the scikit-learn framework. The experiments were performed on a server with a single-core CPU, 2.6 GHz, 2G memory, and Ubuntu 18.04.

**Figure 5:**
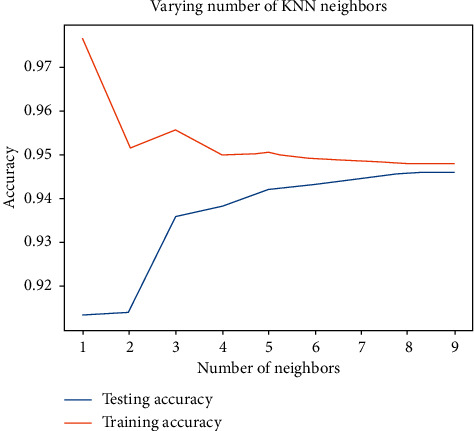
Result of varying number of KNN neighbors.

KNN was performed on the selected dataset first. In order to choose the most appropriate number of neighbors, we trained and tested different numbers of neighbors on the dataset consisting of the data of all 14 vehicles and obtained their accuracy, respectively. The result of varying number of KNN neighbors is shown in Figure 5, from which we can see that the accuracy of the training set decreases as the number of neighbors increases, and at the same time the accuracy of the test set increases as the number of neighbors increases. When the number of neighbors increases to 9, the accuracy of both the training and test sets remains stable. Therefore, 9 is appropriate to be determined as the number of neighbors.

**Figure 6:**
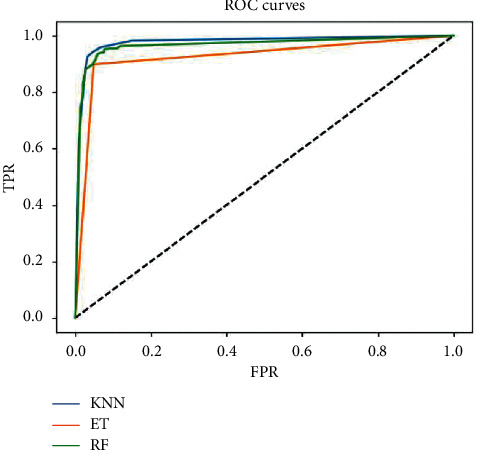
The ROC curves of designed models.

After determining the number of neighbors, KNN model was designed and performed on the dataset consisting of the data of all 14 vehicles, compared with ET and RF. The ROC of designed models including KNN, ET, and RF is shown in Figure 6 and the AUC values of the three models are shown in Table 6. Obviously, from the ROC curves, the performance of KNN is better than RF and obviously better than ET. It can be seen more clearly in Table 6 that the AUC value of KNN is 97.73%, while the AUC values of ET and RF are 92.55% and 96.69%, respectively, showing that the performance of KNN is the best in these three models.

After experimental testing on the dataset of all 14 vehicles, in order to analyze the effect of continuous lane-change detection of vehicles in real scenes, the designed KNN model was used to perform experiments on the respective datasets of the 14 vehicles and compared with ET and RF.

The detailed sample sizes of lane-change behaviour on 14 selected vehicles are shown in Table 7. In the dataset, we can see that, in the real scene, the number of lane-keep samples is generally larger than the number of left and right lane-change samples. Among the 14 vehicles, only the total number of the lane changes to left (LCL) and the lane changes to right (LCR) of the vehicle numbered 2791 is greater than the number of lane-keep (LK), and the number of each item is still less than the number of LK. Besides, there are continuous lane changes in vehicles numbered 2795 and 2825. A single lane change to the left and right is recorded as LCL-1 and LCR-1, while a continuous lane change to the left and right is recorded as LCL-2 and LCR-2, respectively.

**Figure 7:**
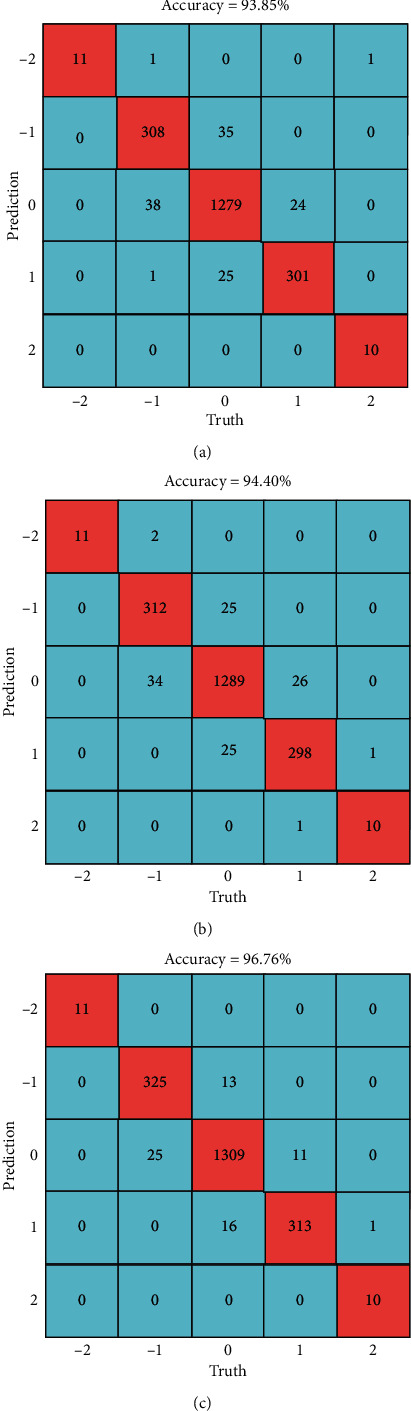
The confusion matrix of detection results: (a) ET, (b) RF, and (c) KNN.

The dataset combining all 14 selected vehicles is divided into training and testing sets according to a ratio of 0.75 to 0.25. The confusion matrix of the detection results is shown in Figure 7, which illustrates that the KNN model has the highest detection accuracy, followed by RF, and ET has the lowest detection accuracy. In addition, detection errors mainly occur between the non-lane-change and the single lane-change behaviour, the probability of false detection between sequential lane-change behaviour and other behaviours is small, and the probability of false detection between left lane-change and right lane-change behaviour is also small. It is worth noting that, in the KNN and RF models, there is no misdetection between the left lane-change, right lane-change, and non-lane-change behaviour.

The experimental results of lane-change detection performed on 14 selected vehicles are shown in Table 8, in which the evaluation indicator mACC denotes mean accuracy of the detection model on all lane-change behaviours. From the experimental results, KNN performed best in the lane-change detection results of all 14 vehicles, while in the lane-change detection results of 14 vehicles, ET performed better than RF in 4 vehicles, RF performed better than ET in 7 vehicles, and ET and RF performed the same in the remaining 3 vehicles. KNN's lane-change detection accuracy ranges from 89.57% to 100%, ET's lane-change detection accuracy ranges from 83.33% to 99.50%, while RF's lane-change detection accuracy ranges from 86.09% to 99.50%. Besides, combined with the sample sizes, in the vehicles with the numbers of 3063 and 2825, the number of samples of the lane-change left and the lane-change right is small, and the detection results are relatively poor, indicating that too few samples will affect the accuracy of the lane-change detection.

## 5. Conclusions

This paper proposed a lane-change detection method for intelligent and connected vehicles. Based on the feature selection of vehicle lane-change behaviour, the detection model based on machine learning was designed, and the effect verification and comparison were performed on the selected dataset. The dataset based on NGSIM Interstate 80 Freeway Dataset was processed for lane-change detection first. After that, feature selection for lane-change detection was performed on the processed dataset, and the lateral velocity was selected as the feature for lane-change detection. Then, the lane-change model was analyzed based on the real data in the processed dataset and the detection model was designed. Finally, the number of KNN neighbors was determined based on experiment, and the performance of KNN, ET, and RF was analyzed by the evaluation indicators. From the experimental results, the designed KNN model performed best in all datasets of the selected 14 vehicles, with detection accuracy ranging from 89.57% to 100%, indicating that it can well complete the task of lane-change behaviour detection for ICVs.

As for future work, the lane-changing scene can be extended by the measured data from the vehicle sensors to establish a more widely adaptable dataset, and the detection model can be further optimized and then implemented on embedded hardware to achieve a lane-change real-time detection system for ICVs.

## Figures and Tables

**Table 1:** The composition of processed data.

Attribute label	Attribute definition
Vehicle_ID	Vehicle identification number.
LX_m	Lateral (*X*) coordinate of the front center of the vehicle in meter with respect to the left-most edge of the section in the direction of travel.
LY_m	Longitudinal (*Y*) coordinate of the front center of the vehicle in meter with respect to the entry edge of the section in the direction of travel.
V_Length	Length of vehicle in feet.
V_Width	Width of vehicle in feet.
Vel_m/s	Instantaneous velocity of vehicle in m/s.
Acc_m/s^2^	Instantaneous acceleration of vehicle in m/s^2^.
Acc_X	Instantaneous lateral acceleration of vehicle in m/s^2^.
Vel_X	Average lateral velocity of vehicle in m/s.
Lane_ID	Current lane position of vehicle.
Lane_change	Current lane-change behaviour of vehicle.

**Table 2:** Precision result of feature selection.

Selected features	KNN	Extra trees (%)	Random forest (%)
LX_m; LY_m	60.28	70.63	74.28
V_Length; V_Width	62.38	65.26	65.29
Vel_m/s	49.14	50.26	50.67
Acc_m/s^2^	43.28	43.62	43.52
Vel_X	94.85	91.73	92.31

**Table 3:** Features ranked based on importance.

Random forest			Extra trees		
Rank	Feature	Importance	Rank	Feature	Importance
1	Vel_X	0.712	1	Vel_X	0.788
2	Vel_m/s	0.094	2	Vel_m/s	0.061
3	Acc_m/s^2^	0.052	3	Acc_m/s^2^	0.042
4	LX_m	0.044	4	LX_m	0.032
5	LY_m	0.040	5	LY_m	0.030
6	V_Length	0.031	6	V_Length	0.024
7	V_Width	0.027	7	V_Width	0.022

**Table 4:** Description of KNN algorithm.

**Input:** Training object (*x*, *y*) ∈*I* and test object $z=\left(\hat{x},\hat{y}\right)$

**Processing:** Compute distance $d=\left(\hat{x},\hat{y}\right)$ between *z* and every object (*x*, *y*) ∈*I*. Select *I*_*z*_ ⊆ *I*, the set of *k* closest training objects to *z*.

**Output:** $\hat{y}=\arg_{v}\max\sum_{\left(x_{i},y_{i}\right)\in I_{z}}F\left(v=y_{i}\right)$

**Table 5:** Confusion matrix of prediction results.

Prediction truth	Positive	Negative
True	True positive (TP)	True negative (TN)
False	False positive (FP)	False negative (FN)

**Table 6:** AUC values of designed models.

	KNN (%)	ET (%)	RF (%)
AUC values	97.73	92.55	96.69

**Table 7:** Sample size of lane-change behaviour on selected vehicles.

ID	Behaviour	Sample size
3365	LCL	131
	LK	444
	LCR	133

3362	LCL	79
	LK	299
	LCR	79

2826	LCL	39
	LK	123
	LCR	39

2804	LCL	45
	LK	267
	LCR	45

2795	LCL-2	36
	LCL-1	46
	LK	613
	LCR-1	48
	LCR-2	35

2782	LCL	152
	LK	935
	LCR	151

2778	LCL	91
	LK	620
	LCR	91

3363	LCL	127
	LK	334
	LCR	127

3063	LCL	23
	LK	99
	LCR	22

2791	LCL	207
	LK	379
	LCR	206

2800	LCL	100
	LK	300
	LCR	99

2825	LCL-2	12
	LCL-1	13
	LK	123
	LCR-1	15
	LCR-2	11

2779	LCL	134
	LK	526
	LCR	135

2774	LCL	100
	LK	379
	LCR	102

**Table 8:** Experimental results of lane-change detection on selected vehicles.

ID	Model	Behaviour	*P* (%)	*R* (%)	*F*1 (%)	mACC (%)
3365	ET	LCL	91	94	93	97.18
		LK	98	97	98	
		LCR	100	100	100	
	RF	LCL	91	94	93	96.61
		LK	97	97	97	
		LCR	100	97	99	
	KNN	LCL	97	97	97	98.87
		LK	99	99	99	
		LCR	100	100	100	

3362	ET	LCL	68	89	77	84.35
		LK	89	84	86	
		LCR	88	82	85	
	RF	LCL	71	89	79	86.09
		LK	91	85	88	
		LCR	89	86	87	
	KNN	LCL	76	100	86	89.57
		LK	97	85	91	
		LCR	87	93	90	

2826	ET	LCL	89	89	89	94.12
		LK	94	97	96	
		LCR	100	86	92	
	RF	LCL	80	89	84	90.20
		LK	92	94	93	
		LCR	100	71	83	
	KNN	LCL	89	89	89	96.08
		LK	97	97	97	
		LCR	100	100	100	

2804	ET	LCL	73	80	76	93.33
		LK	97	94	96	
		LCR	90	100	95	
	RF	LCL	89	80	84	94.44
		LK	97	96	96	
		LCR	82	100	90	
	KNN	LCL	90	90	90	95.55
		LK	97	97	97	
		LCR	89	89	89	

2795	ET	LCL-2	91	100	95	94.36
		LCL-1	67	77	71	
		LK	99	95	97	
		LCR-1	81	100	90	
		LCR-2	100	83	91	
	RF	LCL-2	91	100	95	96.41
		LCL-1	91	77	83	
		LK	99	98	98	
		LCR-1	81	100	90	
		LCR-2	100	83	91	
	KNN	LCL-2	100	100	100	98.46
		LCL-1	100	77	87	
		LK	98	100	99	
		LCR-1	100	100	100	
		LCR-2	100	100	100	

2782	ET	LCL	100	98	99	99.03
		LK	99	100	99	
		LCR	100	95	97	
	RF	LCL	100	98	99	98.71
		LK	98	100	99	
		LCR	100	92	96	
	KNN	LCL	100	98	99	99.68
		LK	100	100	100	
		LCR	100	100	100	

2778	ET	LCL	96	100	98	98.01
		LK	100	97	99	
		LCR	88	100	94	
	RF	LCL	96	100	98	98.01
		LK	100	97	99	
		LCR	88	100	94	
	KNN	LCL	100	100	100	99.00
		LK	99	100	99	
		LCR	100	91	95	
3363	ET	LCL	87	77	82	87.07
		LK	87	90	89	
		LCR	86	89	88	
	RF	LCL	90	74	81	86.39
		LK	86	90	88	
		LCR	83	89	86	
	KNN	LCL	96	77	86	91.16
		LK	90	95	92	
		LCR	90	96	93	

3063	ET	LCL	50	33	40	83.33
		LK	84	96	90	
		LCR	100	40	57	
	RF	LCL	50	33	40	86.11
		LK	87	96	92	
		LCR	100	60	75	
	KNN	LCL	100	67	80	97.22
		LK	97	100	98	
		LCR	100	100	100	

2791	ET	LCL	100	77	87	88.89
		LK	82	98	89	
		LCR	95	87	91	
	RF	LCL	100	84	91	89.90
		LK	84	97	90	
		LCR	93	84	88	
	KNN	LCL	96	85	91	92.42
		LK	89	96	92	
		LCR	96	96	96	

2800	ET	LCL	88	93	90	88.00
		LK	94	85	90	
		LCR	72	90	80	
	RF	LCL	91	97	94	92.00
		LK	96	91	93	
		LCR	82	90	86	
	KNN	LCL	97	97	97	95.20
		LK	97	95	96	
		LCR	86	95	90	

2825	ET	LCL-2	50	100	67	90.91
		LCL-1	50	67	57	
		LK	100	91	95	
		LCR-1	67	100	80	
		LCR-2	100	100	100	
	RF	LCL-2	50	100	67	90.91
		LCL-1	67	67	67	
		LK	100	94	97	
		LCR-1	50	50	50	
		LCR-2	83	100	91	
	KNN	LCL-2	100	100	100	95.45
		LCL-1	100	100	100	
		LK	100	97	98	
		LCR-1	50	100	67	
		LCR-2	100	80	89	

2779	ET	LCL	98	100	99	99.50
		LK	100	99	100	
		LCR	100	100	100	
	RF	LCL	98	100	99	99.50
		LK	100	99	100	
		LCR	100	100	100	
	KNN	LCL	100	100	100	100
		LK	100	100	100	
		LCR	100	100	100	

2774	ET	LCL	91	91	91	94.52
		LK	96	96	96	
		LCR	94	94	94	
	RF	LCL	95	91	93	95.21
		LK	96	97	96	
		LCR	94	94	94	
	KNN	LCL	100	100	100	97.26
		LK	98	98	98	
		LCR	94	94	94	

## Data Availability

The related data are available online at https://github.com/WHUT-DLY/Processed-data-based-on-the-Next-Generation-Simulation-NGSIM-Interstate-80-Freeway-Dataset.
